# Synergistic TLR2/6 and TLR9 Activation Protects Mice against Lethal Influenza Pneumonia

**DOI:** 10.1371/journal.pone.0030596

**Published:** 2012-01-27

**Authors:** Michael J. Tuvim, Brian E. Gilbert, Burton F. Dickey, Scott E. Evans

**Affiliations:** 1 Department of Pulmonary Medicine, The University of Texas MD Anderson Cancer Center, Houston, Texas, United States of America; 2 Center for Infectious and Inflammatory Diseases, Institute of Biosciences and Technology, Texas A&M Health Science Center, Houston, Texas, United States of America; 3 Department of Molecular Virology and Microbiology, Baylor College of Medicine, Houston, Texas, United States of America; College of Medicine, Hallym University, Republic of Korea

## Abstract

Lower respiratory tract infections caused by influenza A continue to exact unacceptable worldwide mortality, and recent epidemics have emphasized the importance of preventative and containment strategies. We have previously reported that induction of the lungs' intrinsic defenses by aerosolized treatments can protect mice against otherwise lethal challenges with influenza A virus. More recently, we identified a combination of Toll like receptor (TLR) agonists that can be aerosolized to protect mice against bacterial pneumonia. Here, we tested whether this combination of synthetic TLR agonists could enhance the survival of mice infected with influenza A/HK/8/68 (H3N2) or A/California/04/2009 (H1N1) influenza A viruses. We report that the TLR treatment enhanced survival whether given before or after the infectious challenge, and that protection tended to correlate with reductions in viral titer 4 d after infection. Surprisingly, protection was not associated with induction of interferon gene expression. Together, these studies suggest that synergistic TLR interactions can protect against influenza virus infections by mechanisms that may provide the basis for novel therapeutics.

## Introduction

Worldwide, lower respiratory tract infections cause more premature death and disability than any other condition [1], [2], [3]. Most years, seasonal influenza pneumonia alone causes more than 40,000 deaths in the United States, despite vaccination programs that have been in place for decades [4], [5]. Pandemic influenzas have even more profound mortality impacts, with more than 50 million influenza-related deaths reported in 1918–9 [6]. The ongoing susceptibility of populations to pandemic influenza was emphasized by the rapid international spread of swine-origin H1N1 influenza in 2009 [7], [8], [9]. Further, the anticipated human-to-human transmission of avian-origin H5N1 influenza, which has already claimed 335 lives worldwide (www.who.int/csr/disease/avian_influenza/) by zoonotic transmission, serves as an obvious indicator of the persisting risk of pandemics [9], [10]. Respiratory viruses, including influenza, have also been characterized as potential agents of bioterror [11].

While a universal influenza vaccine is desirable, efficacy of such a vaccine capable of protecting against future pandemics has not yet been demonstrated [12]. Moreover, it is inevitable in the foreseeable future that populations will remain susceptible to seasonal influenza due to incomplete seasonal vaccination programs [13], [14], [15], [16], epidemiologically-predicted trivalent vaccines that fail to confer immunity to a prevalent strain [17], [18], and host factors that impair initiation or maintenance of vaccine-induced immunity [19], [20], [21].

These concerns led us to investigate whether the intrinsic defense mechanisms of the lungs could be stimulated to broadly protect against pneumonias, independent of vaccine status. We have previously reported that stimulation of lung innate immunity with an aerosolized bacterial lysate could protect against pneumonia caused by bacterial, fungal and viral pathogens, including otherwise lethal influenza A challenges [22], [23], [24], [25], [26], [27]. More recently, we reported that an aerosolized combination of synthetic Toll-like receptor (TLR) agonists could recapitulate the protection conferred by the lysate against bacterial infections [25], [28], leading to the question of whether protection against viral pneumonia could also be achieved using this novel combination of TLR ligands. Here, we report that synthetic ligands for TLR2/6 and TLR9 induce robust protection against lethal influenza pneumonia, including from swine-origin H1N1 influenza.

## Results

### Synergistic TLR2/6 and TLR9 stimulation protects against lethal influenza pneumonia

**Figure 1:**
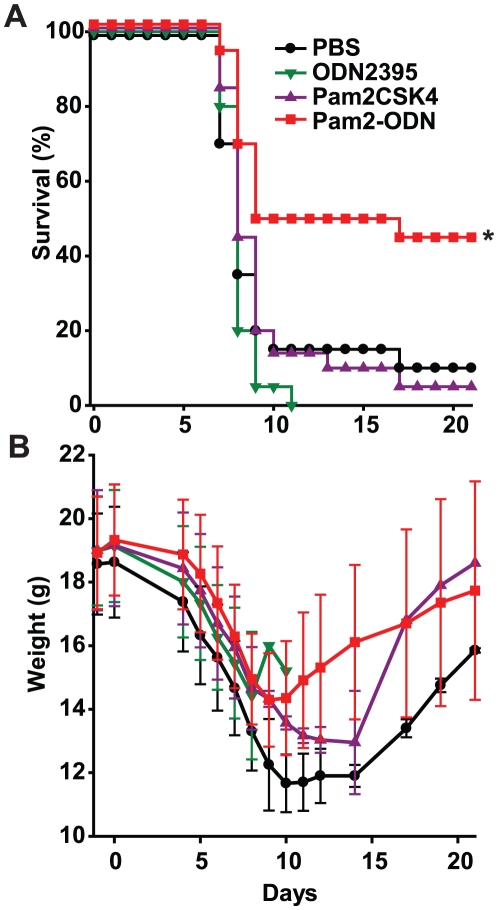
Synergistic TLR stimulation protects against lethal influenza pneumonia, while individual TLR ligands confer no protection. Swiss-Webster mice were challenged with influenza A/Hong Kong/8/68 (H3N2) 24 h after aerosolized treatment with PBS (sham), Pam2, ODN or both (Pam2-ODN). Shown are survival (**A**) and body weight (**B**) of the mice through day 22 after infection (mean ± s.d.). (n = 20 mice/group; *p = 0.03 vs. PBS treated).

Wildtype mice were challenged with a lethal inoculum of influenza A/Hong Kong/8/68 (H3N2) 1 d after a single aerosolized treatment with synthetic TLR ligand(s) or PBS (sham), then observed for 22 d. Treatment of mice with a TLR2/6 agonist (Pam2CSK_4_, “Pam2”) alone or a TLR9 agonist (ODN2395, “ODN”) alone resulted in no protection against lethal influenza pneumonia. However, when both treatments were concurrently delivered (Pam2-ODN) prior to the viral challenge, survival of the infectious challenge was significantly enhanced (Figure 1A). Similarly, while the mean weight loss of mice treated with single ligands did not differ from the infection-related weight loss of sham-treated mice, the mean weight loss of mice treated with Pam2-ODN was significantly less (p<0.05) than that of the sham-treated mice for days 4–14 after infection (Figure 1B). The non-significant weight differences observed after day 14 reflect the recovery phase of just two surviving PBS-treated mice and the heterogeneous recovery rates in the Pam2-ODN treated mice. Intuitively, the weight curves of the surviving Pam2-ODN-treated mice and the PBS-treated mice would be expected to eventually converge at a time beyond the period of observation. As the induced protective effect was substantially greater than additive effect of the individual TLR ligands alone, the Pam2-ODN-induced protection is recognized as synergistic in nature. This is consistent with the synergistic protection we have previously observed in mice pretreated with this TLR ligand combination prior to bacterial challenges [28].

### TLR-induced resistance to influenza pneumonia is inducible before or after infection

**Figure 2:**
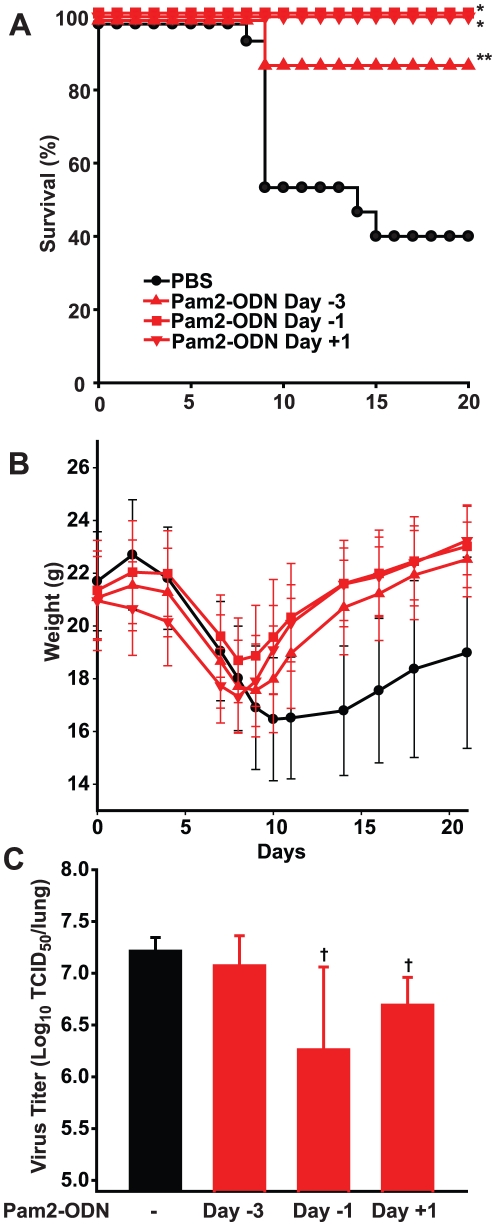
Synergistic TLR stimulation protects against influenza pneumonia whether given before or after infection. Mice were challenged with influenza A following a single aerosolized treatment with Pam2-ODN 3 d before infection, 1 d before infection or 1 d after infection or following a single aerosol treatment with PBS 1 d before infection. Shown are survival (**A**) and body weight (**B**) of the mice through day 22 after infection (mean ± s.d.). Log viral titer of lung homogenates is shown for day 4 after infection for the same groups (**C**, mean ± s.d.). (n = 20 mice group for survival and weight, n – 5 mice/group for lung titers; * p<0.0001 vs. PBS treated, ** p<0.002 vs. PBS treated, † p<0.05 vs. PBS treated).

Our previous studies of inducible resistance indicate that the stimulating treatment can be delivered after the infectious challenge, even when mice are challenged with otherwise lethal inocula of influenza [24], [27], [28]. To determine whether the protective phenomenon of TLR synergy-induced resistance to influenza was restricted to only prophylactic treatment 1 d before viral exposure, mice were challenged after treatment with Pam2-ODN 3 d prior to infection or 1 d after infection, and compared to mice treated 1 d prior to infection or treated with PBS alone. As shown in Figure 2A, survival was significantly enhanced for each Pam2-ODN treatment group compared to PBS-treated mice. Similarly, weight loss was less for all of the Pam2-ODN treated groups by day 10 after infection and this persisted through the end of the observation period (p<0.05, Figure 2B). While the mice treated with Pam2-ODN on the day after viral infection transiently averaged 1–2 g less than the PBS treated mice (days 2 to 7), all three Pam2-ODN treated groups exceeded the mean of the PBS treated mice for most of the observation period. Consistent with our prior observations [27], the greatest induced protection was associated with the greatest reductions in the lung viral titers 4 d after infection (Figure 2C). However, as we have also previously described, the nonsignificant trend towards reduction in viral titer in the mice treated with Pam2-ODN 3 d prior to challenge suggests that early reductions in viral titer are likely only one determinant of the inducible protection. The authors postulate, for example, that treatment may also attenuate the native, injurious inflammatory host response to the virus, may enhance containment of the infection within the lungs, may prevent death due to secondary bacterial infections and may foster effective ongoing viral clearance in addition to rapid induction of pathogen killing. And, much like the cytokine-induced antiviral state is observed in certain leukocytes, it is possible that the TLR stimulus may directly reduce the ability of virus to infect its primary target, the respiratory epithelium.

### TLR3 stimulation does not enhance TLR2/6 and TLR9 protection against influenza pneumonia

**Figure 3:**
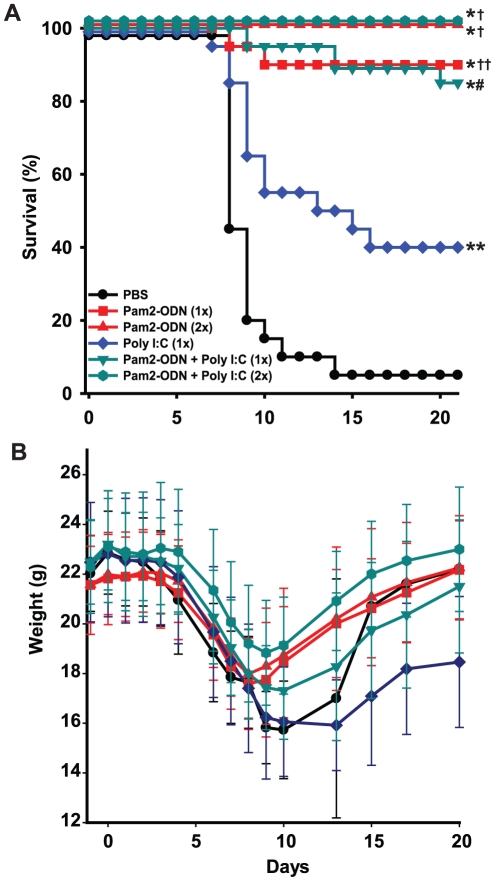
Synergistic TLR2/6 and TLR9 protects against influenza with or without TLR3 stimulation. Mice were challenged with influenza 1 day after a single inhaled treatment with the described treatments. Shown are survival (**A**) and body weight (**B**) of mice through 22 days after challenge (mean ± s.d.). “2x” indicates doubling of the concentration of all TLR ligand components in a corresponding “1x” treatment. (n = 20 mice/group; * p<0.00001 vs. PBS treated, ** p<0.02 vs. PBS treated, † p<0.0001 vs. poly(I∶C) treated, †† p = 0.002 vs. poly(I∶C) treated, # p = 0.004 vs. poly(I∶C) treated).

Treatments that activate TLR3 in mice have been reported to enhance antiviral immunity, including induction of protection against influenza [29], [30], [31], [32], [33]. We tested whether treatment of mice with the TLR3 agonist poly(I∶C) would protect against influenza in our lethal infection model. As shown in Figure 3A, we found that poly(I∶C) treatment delivered 1 d prior to influenza challenge did result in improved survival, supporting the prior literature. However, a single treatment with poly(I∶C) resulted in less protection than a single treatment with Pam2-ODN. Moreover, the addition of poly(I∶C) had no discernable effect on the synergistic response to Pam2-ODN, as the concurrent administration of the TLR3 ligand with the TLR2/6 and TLR9 ligands resulted in no significant differences in survival or body weight. We found that doubling the concentration of Pam2-ODN further improved survival to 100% (Figure 3). At this level of protection, the survival and weight changes of the 1x and 2x Pam2-ODN-treated groups cannot be statistically distinguished from each other. However, it is notable that the increased dose was well tolerated by the mice, inducing neither distress nor worsening survival.

### Protection is not associated with induction of lung interferon expression

Our prior studies of lysate-induced resistance to influenza revealed significant induction of both type I and type II interferon expression [27]. Given prior data suggesting that lung epithelial cells play a critical role in inducible resistance [22], [24], [26], [28], we first assessed Pam2-ODN-induced interferon signaling in MLE-15 lung epithelial cells in isolation. As shown in Table 1, we did not observe the induction of type I, II or III interferon expression by these cells following treatment with Pam2-ODN, despite analyzing identical time points to those studied when investigating lysate-induced protection. While the epithelium appears to play an important role in inducible resistance, we recognized that recruited leukocytes might be the source of the previously observed lysate-induced interferons in the lungs. So, wildtype mice were treated with Pam2-ODN or PBS, and interferon gene expression was analyzed in whole lung homogenates. Again, we did not detect significant induction of interferon genes or known interferon-sensitive antiviral genes by Pam2-ODN treatment, although we did find induction of some interferon receptors. Although there was significant induction of Janus Kinase 1 (JAK1, p<0.01), JAK2 (p<0.00001), and Signal Transducer and Activator of Transcription 1 (STAT1, p<0.00001) expression following PAM2-ODN treatment, pathway analysis did not associate inducible resistance with TLR-enriched interferon signaling (compared to PBS-treated, p = 1.0). To ensure that the absence of interferon-related gene expression was not the result of an insufficient therapeutic stimulus or an insensitive detection technique, we also analyzed interferon-independent, pro-inflammatory cytokine gene expression. Table 2 presents 15 representative examples of these pro-inflammatory cytokines and chemokines that are significantly enriched in the same samples analyzed in Table 1. Unlike the negligible changes seen in the interferon-dependent genes, treatment with Pam2-ODN results in robust expression of IL-6, TNF, IL-1α, IL-1β, and multiple chemokines. In most examples, these findings were observed from both MLE-15 cells in isolation and from whole lung homogenates 4 h after Pam2-ODN treatment.

**Table 1:** Interferon responses to Pam2-ODN.

Symbol	MLE-15 Fold Change	Lung Fold Change	Definition	Accession
*Bak1*	↔	↔	Bcl2-antagonist/killer 1	NM_007523.2
*Bax*	↔	↓ 1.6	Bcl2-associated X protein	NM_007527.2
*Bcl2*	↔	↔	B-cell leukemia/lymphoma 2, variant 2	NM_177410.2
*Gvin1*	↔	↔	GTPase, very large interferon inducible 1, variant B	NM_001039160.2
*Ifi202b*	↔	↔	Interferon activated gene 202B	NM_008327.1
*Ifi203*	↔	↔	Interferon activated gene 203, transcript variant 2	NM_008328.2
*Ifi204*	↔	↔	Interferon activated gene 204	NM_008329.2
*Ifi205*	↔	↔	Interferon activated gene 205	NM_172648.3
*Ifi27*	↔	↔	Interferon alpha-inducible protein 27	NM_029803.1
*Ifi30*	↔	↔	Interferon gamma inducible protein 30	NM_023065.3
*Ifi35*	↔	↓ 1.4	Interferon-induced protein 35	NM_027320.4
*Ifi44*	↔	↔	Interferon-induced protein 44	NM_133871.1
*Ifi47*	↑ 3.3	↑ 2.6	Interferon gamma inducible protein 47	NM_008330.1
*Ifih1*	↔	↔	Interferon induced with helicase C domain 1	NM_027835.1
*Ifit1*	↔	↔	Interferon-induced protein with tetratricopeptide repeats 1	NM_008331
*Ifit2*	↔	↑ 1.9	Interferon-induced protein with tetratricopeptide repeats 2	NM_008332.2
*Ifit3*	↔	↔	Interferon-induced protein with tetratricopeptide repeats 3	NM_010501.2
*Ifitm1*	↔	↔	Interferon induced transmembrane protein 1	NM_026820.2
*Ifitm2*	↔	↑ 1.6	Interferon induced transmembrane protein 2	NM_030694.1
*Ifitm3*	↔	↔	Interferon induced transmembrane protein 3	NM_025378.2
*Ifitm5*	↔	↔	Interferon induced transmembrane protein 5	NM_053088.2
*Ifitm6*	↔	↔	Interferon induced transmembrane protein 6	NM_001033632.1
*Ifitm7*	↔	↔	Interferon induced transmembrane protein 7	NM_028968.1
*Ifna1*	↔	↔	Interferon alpha 1	NM_010502.2
*Ifna2*	↔	↔	Interferon alpha 2	NM_010503.2
*Ifna4*	↔	↔	Interferon alpha 4	NM_010504.1
*Ifna5*	↔	↔	Interferon alpha 5	NM_010505.1
*Ifna6*	↔	↔	Interferon alpha 6	NM_206871.1
*Ifna7*	↔	↔	Interferon alpha 7	NM_008334.2
*Ifna9*	↔	↔	Interferon alpha 9	NM_010507.1
*Ifna11*	↔	↔	Interferon alpha 11	NM_008333.1
*Ifna12*	↔	↔	Interferon alpha 12	NM_177361.2
*Ifna13*	↔	↔	Interferon alpha 13	NM_177347.2
*Ifna14*	↔	↔	Interferon, alpha 14	NM_206975.1
*Ifnab*	↔	↔	Interferon alpha B	NM_008336.2
*Ifnar1*	↔	↔	Interferon alpha and beta receptor 1	NM_010508.1
*Ifnar2*	↔	↑ 3.0	Interferon alpha and beta receptor 2	NM_010509.1
*Ifnb1*	↔	↔	Interferon beta 1, fibroblast	NM_010510.1
*Ifne1*	↔	↔	Interferon epsilon 1	NM_177348.2
*Ifng*	↔	↔	Interferon gamma	NM_008337.1
*Ifngr1*	↔	↑ 1.4	Interferon gamma receptor 1	NM_010511.2
*Ifngr2*	↑ 2.5	↑ 1.7	Interferon gamma receptor 2	NM_008338.2
*Ifnk*	↔	↔	Interferon kappa	NM_199157.2
*Ifnz*	↔	↔	Interferon zeta	NM_197889.1
*Ifrg15*	↔	↔	Interferon alpha responsive gene 15	NM_022329.3
*Igtp*	↔	↑ 1.4	Interferon gamma induced GTPase	NM_018738.3
*Iigp1*	↔	↔	Interferon inducible GTPase 1	NM_021792.3
*Iigp2*	↔	↑ 1.5	Interferon inducible GTPase 2	NM_019440.2
*Isg20*	↔	↔	Interferon-stimulated protein 20	NM_020583.4
*Mx1*	↔	↔	Myxovirus (influenza virus) resistance 1	NM_010846.1
*Oas1a*	↔	↔	2′-5′ oligoadenylate synthetase 1A	NM_145211.1
*Psmb8*	↔	↔	Proteasome subunit beta type 8	NM_010724.1
*Tap1*	↔	↔	Transporter 1, ATP-binding cassette, sub-family B	NM_013683.1

Transcriptional responses of interferon and known interferon-sensitive antiviral genes 4 h after treatment of MLE-15 cells *in vitro* or mouse lungs *in vivo* with Pam2-ODN.

Fold change compares Pam2-ODN-treated samples to PBS-treated samples. ↔ indicates no significant change in gene expression between PBS treated and Pam2-ODN treated samples, ↑ indicates induction of transcription by Pam2-ODN, ↓ indicates repression of transcription by Pam2-ODN.

**Table 2:** Inflammatory cytokine responses to Pam2-ODN.

Symbol	MLE-15 Fold Change	Lung Fold Change	Definition	Accession
*Ccl2*	↔	↑ 33.0	Chemokine (C-C motif) ligand 2	NM_011333.3
*Ccl3*	↔	↑ 53.5	Chemokine (C-C motif) ligand 3	NM_011337.2
*Ccl7*	↔	↑ 15.3	Chemokine (C-C motif) ligand 7	NM_013654.2
*Cx3cl1*	↑ 4.0	↑ 2.4	Chemokine (C-X3-C motif) ligand 1	NM_009142.3
*Cxcl1*	↑ 77.3	↑ 16.6	Chemokine (C-X-C motif) ligand 1	NM_008176.1
*Cxcl2*	↑ 7.9	↑ 14.4	Chemokine (C-X-C motif) ligand 2	NM_009140.2
*Cxcl10*	↑ 1.7	↑ 22.8	Chemokine (C-X-C motif) ligand 10	NM_021274.1
*Cxcl13*	↔	↑ 11.3	Chemokine (C-X-C motif) ligand 13	NM_018866.1
*Cxcl15*	↑ 5.0	↔	Chemokine (C-X-C motif) ligand 15	NM_011339.2
*Cxcl16*	↑ 2.4	↑ 1.3	Chemokine (C-X-C motif) ligand 16	NM_023158.6
*Il1a*	↔	↑ 8.1	Interleukin 1 alpha	NM_010554
*Il1b*	↔	↑ 25.1	Interleukin 1 beta	NM_008361
*Il24*	↑ 3.0	↔	Interleukin 24	NM_053095.1
*Il6*	↔	↑ 2.6	Interleukin 6	NM_031168.1
*Tnf*	↑ 2.0	↑ 24.8	Tumor necrosis factor	NM_013693.1

Transcriptional responses of interferon-independent inflammatory cytokines and chemokines 4 h after treatment of MLE-15 cells *in vitro* or mouse lungs *in vivo* with Pam2-ODN.

Fold change compares Pam2-ODN-treated samples to PBS-treated samples. ↔ indicates no significant change in gene expression between PBS treated and Pam2-ODN treated samples, ↑ indicates induction of transcription by Pam2-ODN, ↓ indicates repression of transcription by Pam2-ODN.

The role of these inflammatory cytokines in inducible resistance remains unclear. IL-6 and TNF were even more robustly induced by the lysate than by Pam2-ODN, but we have shown that they were not required for protection against bacterial pneumonia [24]. Also, given the demonstrations of tachyphylaxis to leukocyte infiltration, but not protection, with repetitive treatments, the induction of these products may represent an epiphenomenon that provides a useful biomarker but may be mechanistically unimportant. However, it is interesting that the four pro-inflammatory cytokines induced by Pam2-ODN 4 h after treatment (IL-1α, IL-1β, IL-6, and TNF) are also the first four pro-inflammatory cytokines induced from respiratory epithelial cells in native influenza infections [34]. While the principal antiviral function of these cytokines is generally presumed to be leukocyte activation, it is conceivable that they also directly shape the epithelial response and that they may eventually prove to be required.

### Class C CpG ODNs synergize most effectively with TLR2/6 agonist to protect against influenza pneumonia

**Figure 4:**
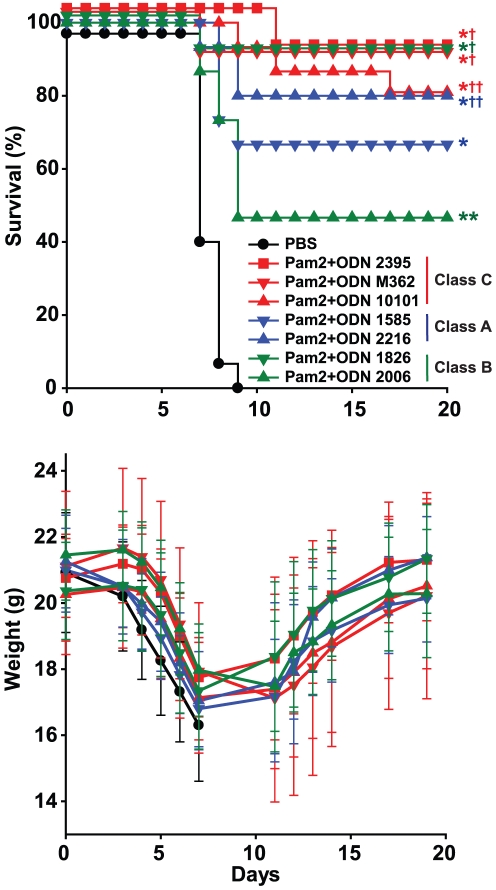
Pam2 treatment synergizes with all classes of TLR9-stimulating CpG oligodeoxynucleotides. Mice were challenged with influenza 1 day after a single inhaled treatment with the described treatments. Shown are survival (**A**) and body weight (**B**) of mice through 22 days after challenge (mean ± s.d.). (n = 20 mice/group; * p<0.00001 vs. PBS treated, ** p = 0.0004 vs. PBS treated, † p = 0.01 vs. Pam2+ODN 2006 treated, † p = 0.1 vs Pam2+ODN 2006 treated).

We have reported that only class C CpG ODNs can effectively activate TLR9 in a manner that synergizes with TLR2/6 to protect against bacterial pneumonia [28]. To test whether class C CpG ODNs are specifically required for TLR-inducible resistance to influenza, we treated groups of mice with one of several aerosolized treatments one day prior to influenza infection. The treatments consisted of Pam2 plus one CpG ODN (class A, B or C) or PBS only, as shown in Figure 4. In contrast to our experience with the bacterial models, all classes of CpG ODNs synergized with Pam2 to protect against influenza pneumonia. As a group, the class C CpG ODNs again protected significantly better non-Class C CpG ODNs (p = 0.025). However, all tested CpG ODNs synergized to some extent with Pam2, and some Class A and Class B CpG ODNs protected as well as Class C ligands when combined with Pam2.

### Synergistic TLR2/6 and TLR9 stimulation protects against swine-origin H1N1 influenza

**Figure 5:**
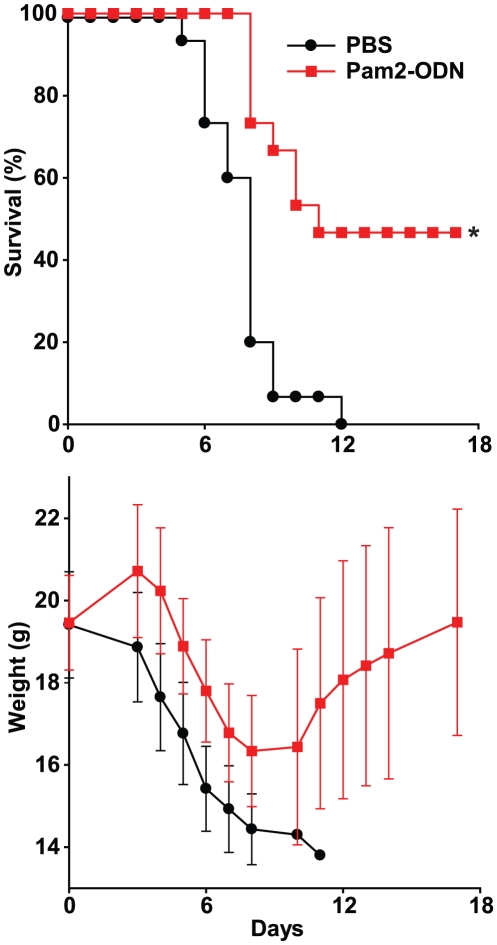
Synergistic TLR stimulation protects against swine-origin H1N1 influenza A pneumonia. Mice were challenged with influenza 1 day after a single inhaled treatment with Pam2 and ODN. Shown are survival (**A**) and body weight (**B**) of mice through 22 days after challenge (mean ± s.d.). (n = 20 mice/group; * p = 0.0004 vs. PBS treated).

To confirm that Pam2-ODN-induced protection was not restricted to a single influenza strain, we tested the ability of Pam2-ODN to protect against highly lethal swine-origin H1N1 influenza. As shown in Figure 5, a single inhaled treatment with Pam2-ODN significantly improved mouse survival of otherwise lethal challenge.

## Discussion

Viral lower respiratory tract infections, particularly those caused by influenza viruses, continue to inflict tremendous annual worldwide mortality [35]. Further compounding this public health urgency are the persisting threat of pandemic influenza infection and an increasing resistance to available antivirals, such as neuraminidase inhibitors [36], [37].

In this study, we find that induction of innate immunity in the lungs with a novel combination of synthetic TLR ligands results in robust protection against otherwise lethal influenza. Consistent with our earlier descriptions of inducible resistance to influenza pneumonia, this protection is generally associated with reductions in the lung viral titers of treated mice [27], though this association is not always observed. Also consistent with our reports of protection induced by treatment of mice with an aerosolized treatment with a bacterial lysate, protection could be induced whether the treatment was applied before or after infection [27].

Type I and II interferon responses have both been identified by other groups as essential to effective antiviral host responses [38], [39]. However, in notable contrast with our observations in lysate-induced resistance to influenza pneumonia [27], we do not find that Pam2-ODN treatment induces significant interferon-related gene expression. As the current studies were not performed in interferon-deficient mice, it is impossible to exclude any role for low-level interferon signaling in Pam2-ODN-induced pneumonia. However, these findings do suggest that reconsideration of the lysate-induced changes in gene expression might be necessary. We previously observed that the lysate induced significant type I and II interferon signaling in the absence of infection, but we also demonstrated that interferon -γ levels in the lungs of infected mice were lower if they had been pretreated with the aerosolized lysate. At that time, we interpreted this to mean that the interferon response was important to inducible resistance, but that the effective interferon response also limited ongoing interferon signaling following clearance of the pathogen. While that interpretation may be correct, the new observation of protection without induction of interferon signaling raises the competing interpretation that interferon signaling is not required for either lysate- or Pam2-ODN-induced resistance to pneumonia. This would be consistent with other prior observations that interleukin-6 and TNF are both profoundly induced by lysate treatment, but are not required for protection against bacterial pneumonias [24].

While these data provide a novel contrast to prior reports of interferon-dependence of the antiviral response, they do not clearly reveal the interferon-independent mechanisms underlying the protection. This is an area of active research, but we have previously reported that Pam2-ODN is capable of inducing expression of both antimicrobial peptides and reactive oxygen species [22], [23], [24], [25], [26], [27], [28], and anticipate that these responses contribute to both inducible viral killing and modulation of untoward elements of the inflammatory response. We have also shown in bacterial infection models that inducible resistance is associated with enhanced containment of pathogens within the lungs [22], [24], [28]. Enhancement of barrier function may contribute to the antiviral response, as well. That this could occur in an interferon-independent manner is supported by recent observations of reactive oxygen species mediated intercellular epithelial antimicrobial communication [40].

Another unexpected finding was the observation that all tested classed of TLR9-stimulating CpG ODNs were capable to some degree of synergizing with Pam2 [28]. Synthetic CpG ODNs can be structurally and functionally categorized into broad classes [41], [42], [43], [44]. Class A ODNs have palindromic sequences on phosphodiester backbones and classically induce secretion of type I and II interferons from leukocytes. Class B ODNs have linear 6-mers on phosphorothioate backbones that induce B cell proliferation and expression of interleukins-6 and -10. Class C ODNs possess characteristics of both A and B classes [43], [45]. These class-specific responses presumably arise from differential endosomal compartmentalization and signaling, with Class A ODNs predominantly promoting IRF-7-mediated signaling from early endosomes and Class B ODNs primarily inducing late endosomal NF-κB activation [43].

We have previously reported that only class C CpG ODNs effectively synergized with the TLR2/6 ligands to protect broadly against bacterial challenges. However, we here clearly demonstrate that class A and class B CpG ODNs can cooperate with Pam2 to protect against influenza viruses, with no discernable statistical difference in the performance of Class A and Class B ligands. This mechanistic difference between the bacterial and viral models will be an area of future investigation. However, as class A and class C, but not class B, CpG ODNs are reported to induce immune responses via interferon signaling, the observation of Pam2 synergy with class B CpG ODNs is consistent with the lack of an essential interferon role.

Because of these differences in Pam2-ODN-induced influenza protection when compared to lysate-induced influenza protection and to Pam2-ODN-induced bacterial protection, it was important to show that this was not a phenomenon that was restricted to a unique viral strain. We excluded that possibility by testing an alternate influenza strain, and demonstrated the effectiveness of this treatment against clinically-relevant pathogens by showing that Pam2-ODN can also protect against swine-origin H1N1 influenza viruses.

This broad, non-toxic host response-focused strategy to preventing influenza-related mortality may provide an opportunity to protect vulnerable populations when vaccines are unavailable or impractical, to contain outbreaks by treating contacts of incident cases, and, potentially, to protect populations that have deficiencies of adaptive immunity.

## Methods

### Animals

Six to eight week old NIH Swiss-Webster mice (Charles River) were used for all experiments. For protection studies, mice were divided into groups of 20 mice (5 for virus lung titers, 15 for survival). All mice were handled in accordance with the policies of the Baylor College of Medicine Institutional Animal Care and Use Committee, full details of the study were approved by that body (approval AN-2307), and any mice that exhibited signs of distress were humanely euthanized.

### Synthetic TLR Ligand aerosol treatment

All treatments were delivered by aerosol. All synthetic TLR ligands were purchased from InvivoGen (San Diego, California), reconstituted in endotoxin-free water, and suspended in 8 ml sterile PBS at indicated concentrations. As we have previously described [22], [23], [27], [28], treatments were aerosolized to the animals for 30 min from an AeroMist CA-209 nebulizer (CIS-US) driven by 10 l/min air supplemented. The nebulizer was connected by polyethylene tubing (30 cm×22 mm) to a 10 liter polyethylene exposure chamber, with an identical efflux tube with a low resistance microbial filter (BB50T, Pall, East Hills, NY) at its end vented to a biosafety hood. Dosing of the TLR ligands was determined by the lowest nebulized concentration required to induce leukocyte infiltration of the lungs, as we have previously reported [28]. Accordingly, the following concentrations were used in the nebulizer reservoir: Pam2 10 µg/ml; poly(I∶C) 100 µg/ml; ODN2395 20 µg/ml. Based on previous experiments, [23], [46] ligand concentrations in the airway lining fluid are calculated to be Pam2 10 ng/ml; poly(I∶C) 100 ng/ml; ODN2395 20 ng/ml. Experiments explicitly using 2X dosing of ligands used double these concentrations. Class comparisons of different CpG ODNs used equimolar concentration of their respective ODN as found in ODN2395 20 µg/ml. Sequences of the tested ODNS were: Class A, ODN 1585 5′-ggggtcaacgttgagggggg-3′ and ODN 2216 5′-gggggacgatcgtcgggggg-3′; Class B, ODN 1826 5′-tccatgacgttcctgacgtt-3′ and ODN 2006 5′-tcgtcgttttgtcgttttgtcgtt-3′; and, Class C, ODN 2395 5′-tcgtcgttttcggcgcgcgccg-3′, ODN M362 5′-tcgtcgtcgttcgaacgacgttgat-3′ and ODN 101015′-tcgtcgttttcgcgcgcgccg-3′.

### Influenza A challenge

A clinical isolate of influenza A/Hong Kong/8/68 (H3N2) (A/HK; Mouse Lung Pool 11-29-05) virus that had been passaged at least nine times through mice was stored as frozen stock (2.8×10^7^ TCID_50_/ml) in the supernatant of mouse lung homogenates [47]. Stock was diluted 1∶300–1∶1,000 in 0.05% gelatin in Eagle's minimal essential medium (Sigma-Aldrich) and aerosolized for 20 min to achieve LD_90_ – LD_100_ (target 100 TDIC_50_/mouse). Viral concentration in the nebulizer before and after aerosolization and in lung homogenates was determined by hemagglutination assay of infected MDCK cells [48]. On day +4, 5 mice from each group were sacrificed and their lungs removed. Lungs were homogenized by beadbeating and the levels of virus determined. Remaining mice in each group were observed daily for up to 21 days for overt illness, morbidity and mortality. Mice were weighed on days 0 and +4, and three times weekly from day +7 until day +21. Influenza A/California/04/2009 (H1N1) was obtained from the Centers for Disease Control and Prevention (Atlanta, GA) as MDCK passage 3 (CDC ID Number 200971204). The virus was grown in MDCK cells [45] and on passage 10, a sucrose purified tissue culture pool was prepared. The 30/50% sucrose interface was collected (passage 11), aliquoted and used for aerosol infection of mice. The titer of the stock virus was 9.8 TCID_50_/mL and was diluted 1∶400 in 0.05% gelatin-MEM before nebulization. The diluted virus was added to the reservoir (9 mL) of an Aerotech II neublizer (CIS-USA, Bedford, MA) flowing at 10 L of air/min and used to treat mice as described above. The targeted dose after 20 min was estimated to be ∼2×10^4^ TCID_50_/mouse.

### Gene expression analysis

To better understand the host response to Pam2-ODN, gene expression microarray analysis was performed after treatment with Pam2, ODN, Pam2-ODN or PBS. For *in vitro* analyses, immortalized mouse distal respiratory epithelial MLE-15 cells [49] were provided by Dr. Jeffrey A. Whitsett, Cincinnati Children's Hospital Medical Center, and grown in monolayer to approximately 80% confluence, then the designated treatments were added to the culture media for 4 h, then the cells were collected by scraping, as previously described [28]. For *in vivo* analyses, wild type mice were exposed by aerosol to the designated treatments, as described above, then euthanized after 4 h for comparison to PBS-treated mice. The lungs were sterilely-resected and mechanically homogenized. Total RNA was isolated from lung homogenates and cell culture samples using the RNeasy system (Qiagen), and cRNA was synthesized and amplified from equal masses of total RNA using the Ilumina TotalPrep RNA amplification kit (Ambion). Amplified cRNA was hybridized and labeled on MouseRef-8 v2.0 Expression BeadChips (Illumina), then scanned on an Illumina iScan. Primary microarray data were deposited at the NCBI Gene Expression Omnibus (http://www.ncbi.nlm.nih.gov/geo/) consistent with MIAME standards (GEO Accession GSE26864, *in vitro*, and GSE28994, *in vivo*). Primary signal intensity was normalized between and within samples, and differentially expressed genes were identified based on signal change and inter-sample variation. Gene ontology analysis was performed using the NIAID Database for Annotation, Visualization and Integrated Discovery (DAVID) and the KEGG Database (GenomeNet). Differentially expressed genes were mapped to signaling pathways using Ingenuity Pathways Analysis 9.0 (Ingenuity Systems), and the pathway nodules were individually reviewed.

To characterize the interferon-related gene expression changes induced by Pam2-ODN, Table 1 presents a list of genes containing all transcripts from the Ingenuity Pathway Analysis canonical interferon signaling pathway, detected interferon-related JAK-STAT-dependent transcripts in KEGG, and additional interferon-sensitive antiviral transcripts identified by the authors. Baseline signal intensity values of 1 were assigned to undetected control transcripts in order to avoid reporting infinite fold change values. Samples treated with single TLR ligands (Pam2 only or ODN only) were analyzed but not presented, as they were not deemed to yield additional information beyond that presented in Table 1. Data from all tested conditions is included in the GEO deposits referenced above.

### Statistical methods

Summary statistics for virus in lung tissue were compared using Student's t-test. Proportions of mice surviving pathogen challenges were compared using Fisher's exact text on the final day of observation, and log-rank comparisons of survival distribution were performed using Kaplan-Meier estimation. Weight comparisons were made between experimental groups using two-tailed Student's t-test for each experimental day. All data shown are representative of at least two independent experiments, and were not combined for analysis because of modest differences in virus challenge doses. Analyses were performed using SAS/STAT (SAS Institute).
